# Standardization and validation of Dot-ELISA assay for *Paracoccidioides brasiliensis* antibody detection

**DOI:** 10.1186/s40409-017-0101-3

**Published:** 2017-02-15

**Authors:** Camila Mika Kamikawa, Rinaldo Poncio Mendes, Adriana Pardini Vicentini

**Affiliations:** 1 Laboratory of Mycosis Immunodiagnosis, Center of Immunology, Adolfo Lutz Institute, Av. Dr. Arnaldo, 355, 11o andar, sala 1117 São Paulo, SP Brazil; 2 Graduate Program in Sciences, Disease Control Coordination of the São Paulo State Health Secretariat, São Paulo, SP Brazil; 3 Department of Tropical Diseases, Botucatu Medical School, São Paulo State University (UNESP – Univ Estadual Paulista), Botucatu, São Paulo State Brazil

**Keywords:** Paracoccidioidomycosis, *Paracoccidioides brasiliensis*, Immunodiagnostic tool, Dot-ELISA, Validation

## Abstract

**Background:**

Paracoccidioidomycosis (PCM) is a neglected systemic mycosis caused by a dimorphic fungus of the *Paracoccidioides* genus. The standard diagnosis is based on isolation of the fungi in culture, and by microscopic visualization of characteristic multiple budding yeast cells in biological samples. However, in some situations, access to the site of injury prevents the collection of biological material. A variety of immuno-serological techniques has proven useful for allowing inferring diagnosis with a certain degree of certainty, thus optimizing time. The aim of this study was to standardize and validate the Dot-ELISA (DE) assay, comparing it with the serological standard, double immunodiffusion (DI).

**Methods:**

In order to standardize the DE assay, 143 serum samples were used. Out of those, 23 were from apparently healthy patients, 77 were from patients with confirmed PCM and 43 were from patients with other lung infections (tuberculosis, aspergillosis and histoplasmosis). To validate the DE technique, 300 serum samples from patients with PCM clinical suspicion (probable and possible cases) were employed, and these results were compared with those of DI.

**Results:**

The DE assay showed sensitivity of 91%, specificity of 95.4%, positive predictive value of 96%, negative predictive value of 98.2%, accuracy of 93%, and great precision (k = 0.93). In addition, the nitrocellulose membranes have proved to be viable for using at least 90 days after *P. brasiliensis* B-339 antigen sensitization.

**Conclusion:**

Dot-ELISA method was found to be an extremely promising tool as serologic screening technique, because of its high sensitivity. Furthermore, Dot-ELISA shows the prospect of being transferred to laboratories of mycoserology including those with fewer resources or even to be used directly in the field. It has an excellent shelf life – membranes coated with antigen can be used for testing without changes in the pattern of reactivity among laboratories – and presents reliable values of sensitivity, specificity, predictive values, accuracy and a high correlation with the serological standard methodology. Based on the present findings, it possible to state that this technique constitutes a remarkable option to be used in routine diagnosis for public health centers.

## Background

Paracoccidioidomycosis (PCM) is the most important systemic mycosis of Latin America caused by the thermally dimorphic fungus *Paracoccidioides* spp. [1–4]. Endemic areas extends from Argentina to Central America; however, approximately 80% of PCM cases, especially due to *Paracoccidioides brasiliensis*, have been reported in Brazil [1, 5]. In Brazil, the disease is considered the eighth cause of death among infectious and parasitic diseases, presenting a mortality rate of 1.4 per million inhabitants [6]. PCM presents a wide spectrum of clinical manifestations and it is more frequent in males than in females (a ratio of approximately 7.2:1) and affects people from 30 to 60 years old, mainly the ones living in rural areas [7–9].

The definitive diagnosis of *P. brasiliensis* infection consists in the direct microscopic examination of biological specimens and their culturing followed by macro and microscopic observation for the fungus identification [10, 11]. However, serological techniques are usually simpler than culture and are employed in early diagnosis of PCM, they are also useful for monitoring its evolution and response to treatment [3, 10–14].

Among different serological assays, double immunodiffusion (DI) is the standard method used for the diagnosis of PCM. DI assay is high specific, but its sensitivity may vary between 65 to 100% depending on the antigenic preparation used [3, 12, 15]. Although the DI assay has advantages regarding cost and feasibility, the implementation of a faster and more sensitive test in the serological routine method is necessary. It could contribute to early initiation of appropriate therapy, which could prevent further damage and help in monitoring fungal dissemination to other organs.

In the past decades, many investigators have described the application of serological tests to the diagnosis of PCM. From the complement fixation test to the immunoenzymatic assays, there has been an increased interest in methodologies that are fast, simple to perform and inexpensive [3, 10, 12]. Dot-ELISA has been widely accepted as a rapid, versatile and simple test based on the principle of enzyme immunoassays, for the detection of many protozoan, virus and fungus diseases [16]. The use of Dot-ELISA for diagnosis of PCM was previously reported by three groups – namely Taborda and Camargo [17], Martins et al. [18] and Correa et al. [19] – without crucial differences in sensitivity and specificity. In all these tests, different reagents and antigenic non-standardized preparations were employed.

In order to improve the serological parameters for the diagnosis of PCM, the present study aimed to produce a fast and accurate method to detect anti-*P. brasiliensis* antibodies employing crude antigen (culture filtrate) and to evaluate the performance and applicability of Dot-ELISA assay as a rapid screening test for the diagnosis of PCM, comparing it to the standard technique, double immunodiffusion.

## Methods

### Serum samples

A study was designed with a total of 443 serum samples obtained from March 2012 to June 2014 at the Laboratory of Mycosis Immunodiagnosis of Adolfo Lutz Institute and Department of Tropical Diseases of Botucatu Medical School, UNESP. Seventy-seven serum samples were from patients previously diagnosed with PCM (proven cases), by finding the etiologic agent in biological samples. Twenty-three serum samples from healthy individuals (normal human serum – NHS), sera from individuals with tuberculosis (n = 20) and sera with DI test confirming histoplasmosis (n = 16) and aspergillosis (n = 8) were used as controls (non-PCM). Three hundred serum samples obtained from patients with “clinical suspicion” (probable and possible cases) of PCM were included in this study. The cases were classified following the Guideline on Paracoccidioidomycosis [20] and EORTC/MSG Consensus Group criteria (European Organization for Research and Treatment of Cancer/Mycosis Study Group) [21]. All samples were obtained from patients residing and domiciled in São Paulo state. Proven cases were from Botucatu region, probable and possible cases were from Campinas, Rio Claro, Sorocaba and São Paulo regions. Anti-*P. brasiliensis* polyclonal antibody, obtained in rabbits, was employed as positive control.

### Ethics consideration

This study was approved by the Research Ethics Committee of Adolfo Lutz Institute, accession number 13673313.1.0000.0059.

### *P. brasiliensis* antigen

The antigen used in this study was 20-day culture filtrate, obtained according to Silva et al. [22], from the yeast phase of B-339 *P. brasiliensis* (ATCC 32069TM) and Pb 113 strains. The fungi were cultured in NGTA – 3% (w/v) neopeptone, 1.8% (w/v) glucose, 0.009% (w/v) asparagine, and 0.125% (w/v) thiamine liquid medium for 20 days at 36 °C with shaking (50 rpm). After incubation time, cultures were treated with an aqueous borate-thimerosal solution (1:5,000), and left to stand for 96 h at 4 °C. After, supernatants were filtered through Whatman n. 1 paper, divided into small volumes and stored at 4 °C until use. Protein contents were subsequently assessed by the Bradford’s method [23].

### Dot-ELISA assay

The methodology was standardized according to the protocols described by Hawkes et al. [24] and Pappas [16] with some modifications. Dot-Elisa for detection of *P. brasiliensis* was performed on nitrocellulose membrane (NC) with 0.22 μm pores (Bio-Rad Laboratories, USA) cut as 1x1 cm squares. The absorption of the *P. brasiliensis* antigens to NC was performed by applying 6 μL of each antigenic preparation in each square, followed by incubation for 30 minutes at 37 °C. After drying, the free binding sites were blocked by 1 h incubation in PBS (pH7.4) containing 5% non-fat dry milk (PBS-L 5%), under continuous shaking at room temperature. These adsorbed and blocked membranes were stored at 8 °C, –20 °C and room temperature until use.

For antibody detection, membranes were placed on 24-well plate and then incubated for 2 h with 500 μL of individual sera from patients with PCM, tuberculosis, histoplasmosis, aspergilosis and sera from healthy individuals diluted either 1:20, 1:40 or 1:100 in PBS containing 3% non-fat dry milk (PBS-L 3%), under continuous shaking at room temperature. After three washes with 500 μL per well of 0.1% Tween-20 in PBS (PBS-T 0.1%), membranes were immersed in solution of secondary antibody, goat anti-human IgG immunoglobulin conjugated with peroxidase (Sigma-Aldrich Co., USA), diluted 1:1,000 or 1:2,000 in PBS-L 3%, for 90 minutes, at room temperature. After incubation, membranes were washed three times again. Then, membranes were immersed in a fresh solution containing 15 mg of 4-chloro-1-naphtol (Sigma-Aldrich Co., USA) diluted in 5 mL of absolute methanol, 30 μL of 30-vol hydrogen peroxide and 20 mL Tris-HCl 0,5 M pH 6.8. The reaction was stopped by washing with distilled water. The NC squares were dried on filter paper and the development of blue dots was considered evidence of a positive result.

### Analysis of the membrane stability

Nitrocellulose membranes previously sensitized with the *P. brasiliensis* antigen, were maintained at –20 °C, 8 °C and room temperature and evaluated progressively for the antigenic reactivity over the time periods of 7, 15, 30, 45, 60 and 90 days, by DE with 11 serum samples randomly chosen among the 300 samples with clinical suspicion of PCM.

Three researchers with expertise in enzyme-linked immunosorbent assays such as immunoblotting (IB) and ELISA performed the analysis of anonymous serum sample. Samples were sequentially numbered avoiding identification of cases and controls by analysts. The DE assays were then performed on different days, including all the steps of the method. The test was carried out in duplicate to evaluate the intra- and inter-assay according to the content.

### Double immunodifusion assay

Reactions were performed according to the modified Ouchterlony’s method [25]. Glass slides were covered with 3.0 mL of 1% agarose gel type II medium (Sigma Chemical Co., USA) diluted in a buffered saline solution pH 6.9 containing 0.4% sodium citrate and 7.5% glycine. Antigen (12 μL) was placed in the central well, while control and patient sera (12 μL) were added in surrounding wells. The slides were incubated in a humid chamber at room temperature for 48 hours. Then, they were washed with saline solution with several changes over a 24-hour period. Gels were dried and stained in 0.4% Coomassie brilliant blue R-250® (Sigma Chemical Co., USA) in an ethanol-acetic acid-water mixture as solvent.

### Statistical analysis

The diagnostic accuracy of DE was evaluated by sensitivity (proportions of positive samples correctly identified by the test) and co-sensitivity (considering DI as the standard assay), specificity (proportions of negative samples correctly identified by the test) and co-specificity (considering DI as the standard assay), predictive values of Dot-ELISA test was determined and the main results were calculated according to Jacobson [26]. The agreement index of tests was calculated and classified by Kappa coefficient according to Landis and Koch [27]. Finally, for proportion comparison we used the chi-square test, performed by the Epi Info 6.1 program (Center for Disease Control and Prevention - http:// www.cdc.gov) with p ≤ 0.05 significance value.

## Results

In order to standardize the Dot-ELISA assay, several experiments were performed to define the optimal conditions to improve this methodology. Conditions established were:

- Employment of culture filtrate obtained from the B-339 strain of *P. brasiliensis* instead of culture filtrate obtained from Pb 113 isolate.
- The optimal concentration of the antigen tested on the NC squares was 42 ng/μL.
- A dilution of 1:100 of sera from patients with paracoccidioidomycosis, tuberculosis, histoplasmosis or aspergilosis and healthy individuals.
- A dilution of 1:2000 of secondary antibody labelled with peroxidase.

**Fig. 1:**
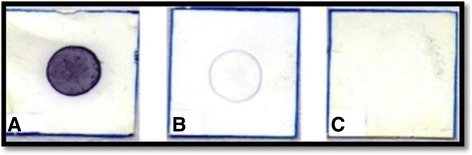
Reactivity pattern of Dot-ELISA assay: (**a**) positive reaction, (**b**) undetermined reaction and (**c**) non-reactive result

Positive reactions were visualized as spots varying from dark purplish blue (strongly reactive sera) to pale purplish blue (weakly reactive sera), depending on the intensity of the reaction. Results were considered negative when no color was observed (Fig. 1). The intrinsic parameters were calculated for both methodologies (DE and DI): sensitivity of 91% (70/77) and 72.7% (56/77), specificity of 95.4% (63/66) and 98.5% (65/66), accuracy of 93% and 84.6% (121/143), positive and negative predictive values were 96% (70/73) and 98.2% (56/57), 90% (63/70) and 75.6% (65/86), respectively (Table 1).

**Table 1:** Serological intrinsic parameters of double immunodiffusion test (DI) and Dot-ELISA technique (DE)

	DI (%)	DE (%)
Sensitivity	72.7	91.0
Specificity	98.5	95.4
Accuracy	84.6	93.0
PPV	98.2	96.0
NPV	75.6	90.0

*PPV* positive predicted value, *NPV* negative predicted value

The performances of DE compared to that of DI standard method were evaluated using 300 samples from patients with clinical suspicion (probable and possible cases) of PCM. Anti-*P. brasiliensis* circulating antibodies were detected in 34% of samples using DI, while DE had detected these antibodies in 47.3% of samples. Discriminatory capacity of both methods suggested that DE assay has a tendency to increase the reactivity pattern, as it was recognized by 40 more samples (13.3%) than DI. Co-positivity (relative sensitivity) and co-negativity (relative specificity) values, using the DI assay as the standard serological test, were 92% (101/110) and 99.5% (189/190) respectively, achieving an agreement level between them of κ = 0.93, considered very good.

The reproducibility of the test has shown that the intra-observer and inter-observer were extremely satisfactory, since their duplicates kept equal both in the result itself (reactive, non-reactive or undetermined) and in the staining intensity.

The evaluation of the viability of previously sensitized nitrocellulose membranes with the B-339 antigen was performed by DE probed against the 11 serum samples from patients with PCM clinical suspicion (probable and possible cases). Reactivity was observed on the membranes up to 90 days.

## Discussion

In Brazil, paracoccidioidomycosis has significant rates of mortality that are estimated to be between 2 and 23% in severe cases, and can reach up to 30% when associated with AIDS [28]. In addition, it is considered the major cause of mortality among mycoses in the country with an average of 111.5 deaths/year [1, 6, 7], according to the Mortality Information System (Brazilian Ministry of Health). The clinical diagnosis of this mycosis can be mistaken for others infectious diseases including those caused by *Leishmania* spp. and *Mycobacterium tuberculosis*. Based on these factors, it becomes clear that the establishment of an early and accurate diagnosis of PCM is necessary for effective and specific initiation of therapy. This measure will reduce the unnecessary use of drugs and empiric therapy, thereby minimizing the emergence of multidrug-resistant fungal strains.

Recently, there has been great interest in the implementation of fast inexpensive methodologies that could also present high rates of sensitivity and specificity in the immunodiagnosis of microbial and parasitic diseases. In this sense, the dot-ELISA assay has been described as an appropriate method for the detection of antibodies or antigens in different human and animal infectious diseases including paracoccidiodomycosis [16–19, 29, 30].

The pioneering study of Taborda and Camargo [17] standardized the methodology for the detection of IgG anti-gp43 antibodies, employing glycosylated and deglycosylated purified gp43, in the diagnosis and follow-up of patients with PCM. The sensitivity and specificity of this assay was 100% when gp43 was treated with sodium metaperiodate. Martins et al. [18] using a mixture composed by culture filtrate and somatic antigens of *P. brasiliensis* yeast cells obtained 96.2% of sensitivity. Correa et al. [19] using the 27 kDa recombinant antigen obtained 100% of sensitivity and 98% of specificity. More recently, Assunção [30] using recombinant gp43 (gp43ΔNt) in PCM immunodiagnosis, observed 100% of sensibility and specificity after sodium metaperiodate treatment.

In the present study, employing culture filtrate obtained from B-339 *P. brasiliensis* strain in DE assay, we obtained 91% of sensitivity and 95.4% of specificity against 72.7% of sensitivity and 98.5% of specificity in DI. Statistical analysis, using chi-squared test, showed that DE technique was significantly more sensitive (p = 0.000067) than DI method, proving and enhancing the applicability of immunoenzymatic assays as optimal methodologies for serological screening.

The small distinct sensitivity observed in this study compared to other results – Taborda and Camargo [17], Martins et al. [18], Correa et al. [19] and Assunção [30] – might be related to differences in the standardization of the methodology, to the nature of the employed antigen, or due the fact that some samples presented no reaction by the DI assay, which indicates that patients were in clinical and/or serological cure. As stated by Perenha-Vianna et al. [31] there is an important limitation on antigenic preparations employed in serological methodologies that must be overcome. *P. brasiliensis* antigens are not commercially available and are usually obtained via in-house productions in research centers. Thus, the lack of standardization in protocols to obtain *P. brasiliensis* antigens causes large differences on test reproducibility.

Moreover, there is some evidence that support the use of crude antigen as immunobiological for anti-*P. brasiliensis* circulating antibody research, instead of 43 kDa purified molecule due to the following: (i) gp43 presents several isoforms [32, 33]; (ii) *P. brasiliensis* expresses variable amounts of gp43 [34]; and (iii) levels of gp43 may vary according to the isolate [35]. Therefore, an individual infected with an isolate that produces gp43 at low rates will not present antibodies specific for this antigen at detectable concentrations, which proves the difficulty in using gp43 for the universal serological diagnosis of PCM [36].

Thus, the present study showed similar intrinsic parameter values of DE assay, using culture filtrate obtained from the B-339 strain of *P. brasiliensis*, when compared to those previously standardized, because it employed more specific and refined antigenic preparations. This finding has major advantages for public health laboratories, since obtaining the culture filtrate is significantly less costly and requires less complex procedures than those required to obtain purified and/or recombinant antigens. Another point that deserves special attention is the good performance (in liters) and the excellent stability of the culture filtrate, more than 15 years after production [37]. Furthermore, we found that the use of culture filtrate also provides good index of sensitivity, specificity, accuracy, positive and negative predictive values, secured the intermediate repeatability and reproducibility of the Dot-ELISA assay.

In recent years, medical laboratories have undergone intense changes, especially regarding quality assurance of products offered. Hawkins [38] highlighted the significant number of publications that link the laboratory failures, estimated at 7-13%, to the possibility of these occurring in the analytical phase. In the validation process, DE methodology was evaluated against 300 convenience samples, i.e. samples from individuals with clinical suspicion (probable and possible cases) of paracoccidioidomycosis. It was found that the relative sensitivity and relative specificity values, using the DI assay as the standard serological test, were 92% and 99.5%, respectively, which achieved an agreement level between them of κ = 0.93, considered great. In the present study, we also verified coherence in the interpretation of results between observers (intra and inter-observer). Comparison of DE methodology with different serologic assays has been thoroughly carried out by other authors [16, 39, 40]. It is unanimous that DE reaction is an excellent tool, primarily for seroepidemiological investigations, that is extremely adaptable in extra-laboratory settings and allows the evaluation of a high number of samples while using smaller amounts of antigen. Another important finding in this study relates to stability of nitrocellulose membranes endowed with the culture filtrate of *P. brasiliensis*.

Despite the good results obtained with membranes that were not blocked and stored at –20 °C, the best results were observed with membranes stored at room temperature up to 90 days (not blocked). Future studies should be conducted to assess the stability for longer periods. However, this finding opens the possibility of sending sensitized membranes to distant laboratories, enabling the realization of seroepidemiological surveys in settlements of people involved with rural activities or living in endemic areas. Historically, DI assay has been the technique of choice in the diagnosis of PCM, immunoenzymatic reactions such as ELISA and IB have also been used for the anti-*P. brasiliensis* antibodies detection [3, 10, 12, 22, 31, 41, 42]. However, despite showing good sensitivity the latter two have low specificity, and often require the previous adsorption of serum samples from patients with paracoccidioidomycosis with *Histoplasma capsulatum* or *Candida albicans*, dilution of serum in galactose or sodium metaperiodate antigen (gp43) treatment [43–45].

The Dot-ELISA proved to be extremely promising, versatile and can be applied as a serological screening test. Because of its high sensitivity as a methodology for the early diagnosis of the disease, it allows the release of negative results in less time than DI assay, i.e. one to seven days, which is of great diagnostic value, especially for the disposal of clinical suspicion of paracoccidioidomycosis. In addition, Dot-ELISA assay is less expensive than ELISA and IB, reagent conservative and does not require electrically powered instruments to give sensitive results. Its portability is ideal for use in field studies where the majority of immunodiagnostic assays are limited by their dependence on electricity, refrigeration or laboratory-grade water.

## ﻿﻿Conclusions

Given the numerous advantages of the presented methodology, this work proposes a broad discussion on the current algorithm recommended by the Epidemiological Surveillance Guide for Paracoccidioidomycosis of São Paulo State, proposing the replacement of indirect ELISA – currently recommended for the screening of serum samples – for Dot-ELISA.

## Abbreviations

DE: Dot-ELISA
DI: double immunodiffusion
IB: immunoblotting
NGTA: neopeptone, glucose, asparagine, thiamine
PCM: paracoccidioidomycosis
